# Hexaaqua­chromium(III) pyridine-2,4,6-tricarboxyl­ate dihydrate

**DOI:** 10.1107/S1600536810023767

**Published:** 2010-06-26

**Authors:** Irfana Mariam, Abdul Rauf, Shahzad Sharif, Islam Ullah Khan, Seik Weng Ng

**Affiliations:** a Department of Chemistry, Government College University, 54000 Lahore, Pakistan; b Department of Chemistry, University of Malaya, 50603 Kuala Lumpur, Malaysia

## Abstract

The chromium(III) atom in the title salt, [Cr(H_2_O)_6_](C_8_H_2_NO_6_)·2H_2_O, has an octa­hedral coordination geometry. In the crystal, the cation, anion and uncoordinated water mol­ecules, both of which are disordered over two positions in a 1:1 ratio, are linked by O—H⋯O hydrogen bonds.

## Related literature

For the crystal structure of hexa­aqua­chromium(III) acetate, see: Eshel & Bino (2001 ▶). For the synthesis of 2,4,6-pyridine­tricarb­oxy­lic acid, see: Syper *et al.* (1980 ▶).
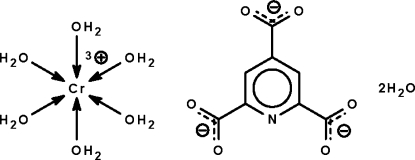


## Experimental

### 

#### Crystal data

[Cr(H_2_O)_6_](C_8_H_2_NO_6_)·2H_2_O
*M* *_r_* = 404.23
Monoclinic, 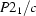

*a* = 7.8610 (3) Å
*b* = 16.9269 (5) Å
*c* = 11.6823 (4) Å
β = 100.649 (1)°
*V* = 1527.70 (9) Å^3^
*Z* = 4
Mo *K*α radiation
μ = 0.83 mm^−1^
*T* = 293 K
0.11 × 0.07 × 0.05 mm

#### Data collection

Bruker Kappa APEXII diffractometer
Absorption correction: multi-scan (*SADABS*; Sheldrick, 1996 ▶) *T*_min_ = 0.915, *T*_max_ = 0.960
14299 measured reflections
3507 independent reflections
2486 reflections with *I* > 2σ(*I*)
*R*_int_ = 0.060

#### Refinement

*R*[*F* ^2^ > 2σ(*F* ^2^)] = 0.050
*wR*(*F* ^2^) = 0.152
*S* = 0.98
3507 reflections
235 parameters
H-atom parameters constrained
Δρ_max_ = 0.64 e Å^−3^
Δρ_min_ = −0.54 e Å^−3^

### 

Data collection: *APEX2* (Bruker, 2009 ▶); cell refinement: *SAINT* (Bruker, 2009 ▶); data reduction: *SAINT*; program(s) used to solve structure: *SHELXS97* (Sheldrick, 2008 ▶); program(s) used to refine structure: *SHELXL97* (Sheldrick, 2008 ▶); molecular graphics: *X-SEED* (Barbour, 2001 ▶); software used to prepare material for publication: *publCIF* (Westrip, 2010 ▶).

## Supplementary Material

Crystal structure: contains datablocks global, I. DOI: 10.1107/S1600536810023767/jh2167sup1.cif

Structure factors: contains datablocks I. DOI: 10.1107/S1600536810023767/jh2167Isup2.hkl

Additional supplementary materials:  crystallographic information; 3D view; checkCIF report

## Figures and Tables

**Table 1:** Selected bond lengths (Å)

Cr1—O1*w*	1.964 (2)
Cr1—O2*w*	1.957 (2)
Cr1—O3*w*	1.941 (2)
Cr1—O4*w*	1.947 (2)
Cr1—O5*w*	1.977 (3)
Cr1—O6*w*	1.952 (3)

**Table 2:** Hydrogen-bond geometry (Å, °)

*D*—H⋯*A*	*D*—H	H⋯*A*	*D*⋯*A*	*D*—H⋯*A*
O1*w*—H11⋯O1	0.84	1.78	2.592 (3)	164
O1*w*—H12⋯O5^i^	0.84	1.93	2.757 (3)	167
O2*w*—H21⋯O2	0.84	1.75	2.565 (3)	164
O2*w*—H22⋯O4^ii^	0.84	1.82	2.662 (3)	177
O3*w*—H31⋯O1^iii^	0.84	1.86	2.670 (3)	164
O3*w*—H32⋯O6^iii^	0.84	1.85	2.667 (3)	163
O4*w*—H41⋯O6^i^	0.84	1.74	2.555 (4)	162
O4*w*—H42⋯O7*w*^iv^	0.84	2.05	2.798 (6)	149
O4*w*—H42⋯O8*w*′^iv^	0.84	1.69	2.448 (7)	149
O5*w*—H51⋯O3^iv^	0.84	2.38	3.070 (5)	140
O5*w*—H52⋯O8*w*^v^	0.84	2.01	2.812 (6)	161
O6*w*—H61⋯O3^ii^	0.84	1.70	2.533 (4)	172
O6*w*—H62⋯O7*w*^vi^	0.84	1.78	2.600 (6)	165
O6*w*—H62⋯O7*w*′^vi^	0.84	2.04	2.780 (7)	148
O7*w*—H72⋯O2	0.84	2.13	2.860 (6)	146
O7*w*—H71⋯O8*w*	0.84	2.32	2.865 (8)	123
O8*w*—H82⋯O4^ii^	0.84	1.83	2.649 (6)	164
O8*w*—H81⋯O5^vii^	0.84	2.06	2.896 (8)	178
O7*w*′—H73⋯O2	0.84	2.02	2.840 (7)	167
O7*w*′—H74⋯O4^ii^	0.84	2.17	2.993 (7)	167
O8*w*′—H83⋯O3	0.84	1.89	2.729 (6)	179
O8*w*′—H84⋯O7*w*′	0.84	1.91	2.744 (9)	171

Symmetry codes: (i) 
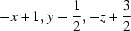
; (ii) 
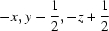
; (iii) 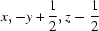; (iv) 
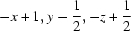
; (v) 
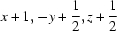
; (vi) 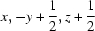; (vii) 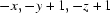.

**Fig. 1.**
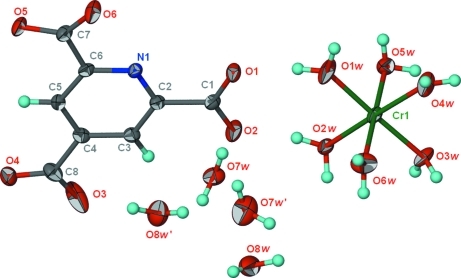
Thermal ellipsoid plot (Barbour, 2001) of [Cr(H2O)6](C8H2NO6).2H2O at the 50% probability level; hydrogen atoms are drawn as spheres of arbitrary radius.

## supplementary crystallographic information

### Comment

The pyridine-2,4,6-tricarboxylate anion is a multifunctional ligand having nitrogen-donor as well as several oxygen-donor sites. Chelation to chromium is expected. However, its reaction with the chromium(III) ion gave instead a salt in which the cation is coordinated by water molecules only (Scheme I, Fig. 1). Interestingly, the only report of a hexaaquachromium carboxylate crystal structure appears to be that of the acetate, an industrially important chemical (Eshel & Bino, 2001). There are no lattice water molecules in the crystal structure.

### Experimental

Pyridine-2,4,6-tricarboxylicacid was prepared by the oxidation of 2,4,6-trimethylpyridine with potassium permanganate(Syper *et al.*, 1980). Chromium chloride hexahydrate (0.03 g, 0.13 mmol) was dissolved in water (10 ml) and this was mixed with the acid (0.11 g, 0.50 mmol) dissolved in water (10 ml). The solution was briefly heated and then set aside for the growth of light purple crystals over several days.

### Refinement

Carbon-bound H-atoms were placed in calculated positions (C–H 0.93 Å) and were included in the refinement in the riding model approximation, with *U*(H) set to 1.2 to 1.5*U*(C).

The two water moleces are both disordered over two positions that, from symmetry considerations, must be in a 1:1 ratio. The water H-atoms were placed in chemically sensible positions on the basis of hydrogen bonding but were not refined (O–H 0.84 Å).

### Crystal data

**Table:**

[Cr(H_2_O)_6_](C_8_H_2_NO_6_)·2H_2_O	*F*(000) = 836
*M**_r_* = 404.23	*D*_x_ = 1.758 Mg m^−^^3^
Monoclinic, *P*2_1_/*c*	Mo *K*α radiation, λ = 0.71073 Å
Hall symbol: -P 2ybc	Cell parameters from 2431 reflections
*a* = 7.8610 (3) Å	θ = 2.6–24.6°
*b* = 16.9269 (5) Å	µ = 0.83 mm^−^^1^
*c* = 11.6823 (4) Å	*T* = 293 K
β = 100.649 (1)°	Prism, purple
*V* = 1527.70 (9) Å^3^	0.11 × 0.07 × 0.05 mm
*Z* = 4	

### Data collection

**Table:**

Bruker Kappa APEXII diffractometer	3507 independent reflections
Radiation source: fine-focus sealed tube	2486 reflections with *I* > 2σ(*I*)
graphite	*R*_int_ = 0.060
φ and ω scans	θ_max_ = 27.5°, θ_min_ = 2.1°
Absorption correction: multi-scan (*SADABS*; Sheldrick, 1996)	*h* = −10→10
*T*_min_ = 0.915, *T*_max_ = 0.960	*k* = −21→21
14299 measured reflections	*l* = −15→14

### Refinement

**Table:**

Refinement on *F*^2^	Primary atom site location: structure-invariant direct methods
Least-squares matrix: full	Secondary atom site location: difference Fourier map
*R*[*F*^2^ > 2σ(*F*^2^)] = 0.050	Hydrogen site location: inferred from neighbouring sites
*wR*(*F*^2^) = 0.152	H-atom parameters constrained
*S* = 0.98	*w* = 1/[σ^2^(*F*_o_^2^) + (0.0867*P*)^2^ + 0.7924*P*] where *P* = (*F*_o_^2^ + 2*F*_c_^2^)/3
3507 reflections	(Δ/σ)_max_ = 0.001
235 parameters	Δρ_max_ = 0.64 e Å^−^^3^
0 restraints	Δρ_min_ = −0.54 e Å^−^^3^

### Fractional atomic coordinates and isotropic or equivalent isotropic displacement parameters (Å^2^)

**Table:**

	*x*	*y*	*z*	*U*_iso_*/*U*_eq_	Occ. (<1)
Cr1	0.54053 (7)	0.15648 (3)	0.39171 (4)	0.02536 (19)	
O1	0.4502 (3)	0.36658 (14)	0.53968 (19)	0.0282 (6)	
O2	0.3235 (4)	0.37338 (16)	0.3540 (2)	0.0408 (7)	
O3	−0.0565 (4)	0.6012 (2)	0.2197 (2)	0.0684 (11)	
O4	−0.1258 (3)	0.68517 (15)	0.3489 (2)	0.0386 (7)	
O5	0.2467 (3)	0.66964 (13)	0.7612 (2)	0.0307 (6)	
O6	0.3712 (4)	0.55364 (15)	0.8057 (2)	0.0395 (7)	
O1w	0.5769 (4)	0.22599 (15)	0.5287 (2)	0.0506 (9)	
H11	0.5517	0.2740	0.5268	0.076*	
H12	0.6408	0.2148	0.5921	0.076*	
O2w	0.4111 (3)	0.23657 (14)	0.29037 (19)	0.0293 (6)	
H21	0.3912	0.2786	0.3235	0.044*	
H22	0.3194	0.2204	0.2483	0.044*	
O3w	0.5096 (3)	0.08414 (13)	0.26061 (19)	0.0313 (6)	
H31	0.4765	0.1051	0.1954	0.047*	
H32	0.4470	0.0448	0.2675	0.047*	
O4w	0.6735 (4)	0.07453 (15)	0.4853 (2)	0.0419 (7)	
H41	0.6514	0.0580	0.5487	0.063*	
H42	0.7368	0.0418	0.4592	0.063*	
O5w	0.7577 (3)	0.19559 (15)	0.3495 (2)	0.0378 (6)	
H51	0.7938	0.1565	0.3170	0.057*	
H52	0.8217	0.2030	0.4145	0.057*	
O6w	0.3271 (4)	0.11722 (18)	0.4342 (2)	0.0448 (7)	
H61	0.2418	0.1082	0.3810	0.067*	
H62	0.2997	0.1085	0.4991	0.067*	
N1	0.3130 (3)	0.50687 (15)	0.5851 (2)	0.0211 (6)	
C1	0.3554 (4)	0.39985 (19)	0.4552 (3)	0.0236 (7)	
C2	0.2751 (4)	0.47842 (18)	0.4768 (3)	0.0216 (7)	
C3	0.1641 (4)	0.5174 (2)	0.3883 (3)	0.0245 (7)	
H3	0.1417	0.4968	0.3132	0.029*	
C4	0.0871 (4)	0.58741 (19)	0.4134 (3)	0.0234 (7)	
C5	0.1272 (4)	0.61709 (19)	0.5264 (3)	0.0232 (7)	
H5	0.0778	0.6639	0.5462	0.028*	
C6	0.2426 (4)	0.57513 (18)	0.6089 (3)	0.0215 (7)	
C7	0.2909 (4)	0.60201 (19)	0.7341 (3)	0.0250 (7)	
C8	−0.0413 (5)	0.6290 (2)	0.3203 (3)	0.0308 (8)	
O7w	0.2400 (7)	0.4369 (3)	0.1235 (4)	0.0362 (12)	0.50
H71	0.1349	0.4321	0.1275	0.054*	0.50
H72	0.3062	0.4247	0.1859	0.054*	0.50
O8w	−0.0236 (8)	0.3188 (3)	0.0680 (5)	0.0536 (16)	0.50
H81	−0.0888	0.3236	0.1171	0.080*	0.50
H82	0.0266	0.2751	0.0814	0.080*	0.50
O7w'	0.1532 (10)	0.3598 (4)	0.1183 (6)	0.0642 (19)	0.50
H73	0.2043	0.3716	0.1857	0.096*	0.50
H74	0.1374	0.3107	0.1159	0.096*	0.50
O8w'	0.0632 (8)	0.5126 (4)	0.0562 (5)	0.0511 (15)	0.50
H83	0.0262	0.5402	0.1062	0.077*	0.50
H84	0.0855	0.4667	0.0819	0.077*	0.50

### Atomic displacement parameters (Å^2^)

**Table:**

	*U*^11^	*U*^22^	*U*^33^	*U*^12^	*U*^13^	*U*^23^
Cr1	0.0390 (3)	0.0177 (3)	0.0159 (3)	0.0005 (2)	−0.0040 (2)	−0.0007 (2)
O1	0.0399 (14)	0.0225 (12)	0.0204 (12)	0.0080 (10)	0.0004 (10)	−0.0002 (9)
O2	0.0606 (18)	0.0325 (14)	0.0237 (13)	0.0142 (13)	−0.0072 (12)	−0.0098 (11)
O3	0.078 (2)	0.105 (3)	0.0187 (14)	0.061 (2)	0.0003 (14)	0.0002 (15)
O4	0.0399 (15)	0.0322 (14)	0.0374 (15)	0.0130 (12)	−0.0090 (12)	−0.0016 (12)
O5	0.0469 (16)	0.0169 (12)	0.0250 (12)	0.0030 (10)	−0.0022 (11)	−0.0040 (9)
O6	0.0665 (19)	0.0258 (13)	0.0215 (12)	0.0134 (12)	−0.0041 (12)	−0.0021 (10)
O1w	0.094 (2)	0.0261 (14)	0.0208 (13)	0.0157 (14)	−0.0177 (14)	−0.0072 (11)
O2w	0.0361 (14)	0.0213 (12)	0.0255 (12)	0.0018 (10)	−0.0077 (10)	−0.0021 (10)
O3w	0.0541 (16)	0.0202 (12)	0.0169 (11)	−0.0106 (11)	−0.0007 (11)	−0.0009 (9)
O4w	0.074 (2)	0.0245 (14)	0.0222 (12)	0.0123 (13)	−0.0027 (13)	0.0049 (10)
O5w	0.0334 (14)	0.0268 (14)	0.0489 (16)	−0.0008 (11)	−0.0039 (12)	−0.0021 (12)
O6w	0.0473 (17)	0.061 (2)	0.0250 (13)	−0.0072 (14)	0.0043 (12)	0.0031 (13)
N1	0.0249 (14)	0.0167 (13)	0.0201 (13)	−0.0004 (10)	0.0002 (11)	0.0011 (10)
C1	0.0268 (17)	0.0210 (16)	0.0221 (16)	−0.0021 (13)	0.0022 (13)	−0.0014 (13)
C2	0.0234 (16)	0.0192 (16)	0.0207 (15)	−0.0014 (13)	0.0005 (13)	0.0025 (12)
C3	0.0247 (17)	0.0285 (18)	0.0186 (15)	0.0015 (14)	−0.0001 (13)	−0.0018 (13)
C4	0.0233 (16)	0.0245 (17)	0.0212 (16)	−0.0009 (13)	0.0013 (13)	0.0063 (13)
C5	0.0235 (16)	0.0195 (16)	0.0262 (16)	0.0015 (13)	0.0032 (13)	−0.0005 (13)
C6	0.0232 (16)	0.0190 (16)	0.0210 (16)	−0.0056 (12)	0.0009 (13)	0.0001 (12)
C7	0.0310 (18)	0.0204 (16)	0.0221 (16)	−0.0020 (14)	0.0012 (13)	−0.0009 (13)
C8	0.0286 (18)	0.038 (2)	0.0249 (18)	0.0067 (15)	0.0019 (14)	0.0095 (15)
O7w	0.038 (3)	0.041 (3)	0.027 (3)	−0.005 (2)	0.001 (2)	0.002 (2)
O8w	0.052 (4)	0.036 (3)	0.063 (4)	0.005 (3)	−0.016 (3)	0.002 (3)
O7w'	0.095 (6)	0.051 (4)	0.046 (4)	0.003 (4)	0.012 (4)	0.008 (3)
O8w'	0.059 (4)	0.048 (4)	0.050 (3)	0.004 (3)	0.019 (3)	−0.006 (3)

### Geometric parameters (Å, °)

**Table:**

Cr1—O1w	1.964 (2)	O6w—H61	0.8391
Cr1—O2w	1.957 (2)	O6w—H62	0.8385
Cr1—O3w	1.941 (2)	N1—C6	1.332 (4)
Cr1—O4w	1.947 (2)	N1—C2	1.335 (4)
Cr1—O5w	1.977 (3)	C1—C2	1.513 (5)
Cr1—O6w	1.952 (3)	C2—C3	1.390 (4)
O1—C1	1.255 (4)	C3—C4	1.386 (5)
O2—C1	1.246 (4)	C3—H3	0.9300
O3—C8	1.251 (5)	C4—C5	1.392 (4)
O4—C8	1.240 (4)	C4—C8	1.514 (4)
O5—C7	1.254 (4)	C5—C6	1.391 (4)
O6—C7	1.254 (4)	C5—H5	0.9300
O1w—H11	0.8363	C6—C7	1.512 (4)
O1w—H12	0.8369	O7w—H71	0.8401
O2w—H21	0.8381	O7w—H72	0.8400
O2w—H22	0.8398	O8w—H81	0.8400
O3w—H31	0.8370	O8w—H82	0.8400
O3w—H32	0.8395	O7w'—H73	0.8400
O4w—H41	0.8393	O7w'—H74	0.8399
O4w—H42	0.8390	O8w'—H83	0.8400
O5w—H51	0.8387	O8w'—H84	0.8400
O5w—H52	0.8387		
			
O3w—Cr1—O4w	88.26 (11)	Cr1—O6w—H62	131.7
O3w—Cr1—O6w	89.85 (11)	H61—O6w—H62	109.5
O4w—Cr1—O6w	90.70 (13)	C6—N1—C2	118.8 (3)
O3w—Cr1—O2w	89.10 (10)	O2—C1—O1	124.8 (3)
O4w—Cr1—O2w	176.95 (11)	O2—C1—C2	117.2 (3)
O6w—Cr1—O2w	90.82 (11)	O1—C1—C2	118.0 (3)
O3w—Cr1—O1w	177.52 (10)	N1—C2—C3	122.2 (3)
O4w—Cr1—O1w	89.26 (11)	N1—C2—C1	116.6 (3)
O6w—Cr1—O1w	89.96 (13)	C3—C2—C1	121.2 (3)
O2w—Cr1—O1w	93.38 (10)	C4—C3—C2	119.2 (3)
O3w—Cr1—O5w	90.16 (11)	C4—C3—H3	120.4
O4w—Cr1—O5w	88.84 (12)	C2—C3—H3	120.4
O6w—Cr1—O5w	179.54 (12)	C3—C4—C5	118.6 (3)
O2w—Cr1—O5w	89.63 (10)	C3—C4—C8	120.3 (3)
O1w—Cr1—O5w	90.00 (13)	C5—C4—C8	121.1 (3)
Cr1—O1w—H11	124.3	C6—C5—C4	118.5 (3)
Cr1—O1w—H12	124.0	C6—C5—H5	120.8
H11—O1w—H12	110.1	C4—C5—H5	120.8
Cr1—O2w—H21	115.4	N1—C6—C5	122.8 (3)
Cr1—O2w—H22	115.0	N1—C6—C7	115.0 (3)
H21—O2w—H22	109.5	C5—C6—C7	122.1 (3)
Cr1—O3w—H31	115.0	O5—C7—O6	123.8 (3)
Cr1—O3w—H32	114.5	O5—C7—C6	119.1 (3)
H31—O3w—H32	109.5	O6—C7—C6	117.0 (3)
Cr1—O4w—H41	123.8	O4—C8—O3	125.3 (3)
Cr1—O4w—H42	124.0	O4—C8—C4	118.8 (3)
H41—O4w—H42	109.5	O3—C8—C4	115.8 (3)
Cr1—O5w—H51	103.2	H71—O7w—H72	112.7
Cr1—O5w—H52	103.1	H81—O8w—H82	106.5
H51—O5w—H52	109.4	H73—O7w'—H74	108.0
Cr1—O6w—H61	118.7	H83—O8w'—H84	110.0
			
C6—N1—C2—C3	−0.3 (5)	C2—N1—C6—C5	1.9 (5)
C6—N1—C2—C1	−178.7 (3)	C2—N1—C6—C7	179.1 (3)
O2—C1—C2—N1	−178.7 (3)	C4—C5—C6—N1	−1.7 (5)
O1—C1—C2—N1	0.5 (5)	C4—C5—C6—C7	−178.8 (3)
O2—C1—C2—C3	2.9 (5)	N1—C6—C7—O5	171.9 (3)
O1—C1—C2—C3	−177.8 (3)	C5—C6—C7—O5	−10.8 (5)
N1—C2—C3—C4	−1.3 (5)	N1—C6—C7—O6	−9.4 (5)
C1—C2—C3—C4	177.0 (3)	C5—C6—C7—O6	167.8 (3)
C2—C3—C4—C5	1.4 (5)	C3—C4—C8—O4	170.1 (3)
C2—C3—C4—C8	−176.9 (3)	C5—C4—C8—O4	−8.2 (5)
C3—C4—C5—C6	0.0 (5)	C3—C4—C8—O3	−7.7 (5)
C8—C4—C5—C6	178.3 (3)	C5—C4—C8—O3	174.0 (4)

### Hydrogen-bond geometry (Å, °)

**Table:**

*D*—H···*A*	*D*—H	H···*A*	*D*···*A*	*D*—H···*A*
O1w—H11···O1	0.84	1.78	2.592 (3)	164
O1w—H12···O5^i^	0.84	1.93	2.757 (3)	167
O2w—H21···O2	0.84	1.75	2.565 (3)	164
O2w—H22···O4^ii^	0.84	1.82	2.662 (3)	177
O3w—H31···O1^iii^	0.84	1.86	2.670 (3)	164
O3w—H32···O6^iii^	0.84	1.85	2.667 (3)	163
O4w—H41···O6^i^	0.84	1.74	2.555 (4)	162
O4w—H42···O7w^iv^	0.84	2.05	2.798 (6)	149
O4w—H42···O8w'^iv^	0.84	1.69	2.448 (7)	149
O5w—H51···O3^iv^	0.84	2.38	3.070 (5)	140
O5w—H52···O8w^v^	0.84	2.01	2.812 (6)	161
O6w—H61···O3^ii^	0.84	1.70	2.533 (4)	172
O6w—H62···O7w^vi^	0.84	1.78	2.600 (6)	165
O6w—H62···O7w'^vi^	0.84	2.04	2.780 (7)	148
O7w—H72···O2	0.84	2.13	2.860 (6)	146
O7w—H71···O8w	0.84	2.32	2.865 (8)	123
O8w—H82···O4^ii^	0.84	1.83	2.649 (6)	164
O8w—H81···O5^vii^	0.84	2.06	2.896 (8)	178
O7w'—H73···O2	0.84	2.02	2.840 (7)	167
O7w'—H74···O4^ii^	0.84	2.17	2.993 (7)	167
O8w'—H83···O3	0.84	1.89	2.729 (6)	179
O8w'—H84···O7w'	0.84	1.91	2.744 (9)	171

Symmetry codes: (i) −*x*+1, *y*−1/2, −*z*+3/2; (ii) −*x*, *y*−1/2, −*z*+1/2; (iii) *x*, −*y*+1/2, *z*−1/2; (iv) −*x*+1, *y*−1/2, −*z*+1/2; (v) *x*+1, −*y*+1/2, *z*+1/2; (vi) *x*, −*y*+1/2, *z*+1/2; (vii) −*x*, −*y*+1, −*z*+1.
